# Decision-making pathways for contraceptive use among refugee and host populations in Adjumani district, Uganda; an exploratory study

**DOI:** 10.1186/s12905-024-03272-z

**Published:** 2024-07-26

**Authors:** Roselline Achola, Christopher Garimoi Orach, Elizabeth Nabiwemba, Lynn M. Atuyambe

**Affiliations:** Department of Community Health and Behavioural Sciences, School of Public Health, College of Health Sciences, Makerere University, P.O Box 7072, Kampala, Uganda

**Keywords:** Contraception, Decision-making, Pathways, Refugees, Host, Processes

## Abstract

**Introduction:**

Contraceptive use is known to have a positive impact on maternal and child health outcomes; however, its use is still low in low-income countries, especially among people in humanitarian situations. This study explored decision-making processes towards the use of contraceptives by people in humanitarian situations to inform program design and uptake.

**Methods:**

A qualitative exploratory study was conducted among women of reproductive age (15–49 years) and men (15–60 years) living in three refugee settlements of Pagirinya, Nyumanzi, and Mirieyi and the surrounding host communities in Adjumani district, Uganda. Data were collected using 49 in-depth interviews (IDIs), 11 Key Informant Interviews (KIIs,) and 20 Focus Group Discussions (FGDs). Inductive thematic analysis was done with the aid of Atlas ti. Version 14.

**Results:**

We found that the decision-making processes entailed linear and nonlinear internalized cognitive and contextual processes involving four dynamic pathways. In the linear pathway, participants reported starting with 1) idea inception, 2) followed by cognitive processing, 3) consultation, and 4) decision-making for contraceptive use. The complex linear pathway happened when participants did not go through consultation but went straight to decision-making. However, participants who followed the non-linear pathway repeatedly went back to cognitive processing. Some women after consultation, or those already using and those not using contraceptives, decided to go back to cognitive processing to reconsider their current positions. This study found that some women who were not using contraceptives ended up using, while some who were using contraception ended up dropping out.

**Conclusions:**

This study showed dynamic decision-making processes involving both internal and external environments as triggers to decision-making for contraceptive use. Interventions to increase contraceptive use should target both users and significant others who influence the decision to use particularly among refugees.

**Trial registration:**

This study was registered by Makerere University School of Public Health Higher Degrees Research and Ethic Committee (HDREC) #188 and approved by Uganda National Council of Science and Technology on 15th/7/2021, Registration number—SS809ES.

## Background

Worldwide, an estimated 89.3 million people have been displaced from their homes by conflict or persecution [1]. Seventy six percent of these are refugees from low- and middle-income countries mainly in sub-Saharan Africa. Uganda has an estimated 1.5 million refugees in designated places (refugee settlements) in the country [1]. In this study, refugees refer to people who have suffered human rights violations and who have fled across the borders of their home countries to seek protection elsewhere [2]. While in displacement, the socio-cultural norms that affect how they live and adapt to the new environment as well as accessing contraceptive services include financial hardship, geographical location, decision making ability and language barrier [3]. Although some women and girls would like to access contraceptives, they have challenges with decision-making due to pressure, coercion, Patriarchism, misconceptions among others [3, 4]. While access to contraceptive services is crucial during displacement, it still remains a challenge for refugees with limited funding and prioritization despite the increasing humanitarian situation [1]. Access to contraception is crucial because the women of reproductive age require to prevent unintended pregnancies that may occur due to sexual violence and would put their lives in jeopardy if they don’t access it [5, 6].

Furthermore, access to contraception in the host country is also influenced by limited knowledge, and unclear decision-making processes for contraceptive use including gender roles and partnerships, social norms, family conflict and disintegration [7]. The drivers of decision-making for contraceptive use include health benefits, social support, economic benefits, and gender dynamics [8]. Barriers to decision-making for contraceptive use include beliefs affecting contraceptive use, cultural norms related to contraceptive use, economic difficulties, health concerns, myths and misconception about contraception, and the price paid for deciding to use contraceptives without permission from family members [3, 4].

Contraception is important because it may save life of a mother by preventing unplanned pregnancies and unsafe abortions as well as reducing the risk of children dying [9–14]. Several studies show that children born within 24 months of an elder sibling have a 60% increased risk of dying before their first birthday compared to those born after 2–3 years with only a 10% risk [11].

Although it is known that a woman’s ability to space and limit her pregnancies has a direct impact on her health and well-being, contraceptive use is still low at 34% in the least developed countries and particularly among refugees [15–17]. In Uganda, Contraceptive Prevalence Rate (CPR) stands at 39% for all methods and 35% for modern contraception. The unmet need for Family Planning is 25% and maternal mortality ratio is 336/100,000 live births [18]. In Adjumani district, the CPR is 25%, far below the national statistics with a high-unmet need for contraception at 25% [18].

In a study done by Achola and others in the same setting revealed that contraceptive use among refugees was 28.2% in Adjumani district [19]. In another study done by Bakessiima and others among the refugee adolescents in northern Uganda revealed that 7.8% were using contraception [20]. However, in a comparative study that was done among refugee and host populations in Kiryadongo district revealed that contraceptive use by refugees was at 71.6% while that of the host population was at 95.5% [21]. This may be high because the refugees in this district come from different countries with various cultural norms as compared to the refugees in Adjumani who are mainly from South Sudan. Thus, the CPR for refugees is always lower than that of the host even in circumstances where use of contraception is presumably high.

In a study conducted in Sub Saharan Africa, it was also noted that only 10% of the refugee women have access to contraception and the remaining 90% have continued to face challenges related to use of contraceptives [22]. These challenges are associated with intimate partner violence, separation, or divorce if they are not authorized by the family members leading to unclear decision-making processes [23, 24].

Whereas contraceptive programs are underpinned by the understanding that women will make decisions to use contraception, the decision-making processes resulting into this outcome is largely un-explored. In a review of literature [25], it was revealed that most studies focused on the current use of contraception and discussed in general the barriers to contraceptive use with a bias on behavior change and not the deeper analysis of what happened during the process of making decisions. Secondly, some scholars have studied systems decision making which includes decision-making units and decision makers [26–29] and not clearly known how individuals make decisions for contraceptive use in humanitarian settings. There is also limited explanatory research on why and how decision-making and other factors continue to influence use of contraception in humanitarian settings. This study, therefore, aimed at exploring the decision-making processes for contraceptive use among refugees and host populations in Adjumani district, Uganda. Beyond the enablers and barriers to contraception use, understanding the decision-making pathways which result into contraceptive use has implications for program design and delivery. Therefore, the findings of this study will guide policy formulation to improve contraceptive services for both refugee and host communities in refugee affected settings.

## Methods

### Study design and setting

A qualitative study was conducted to explore decision-making processes among refugees and host populations in Adjumani district, Uganda. The study participants were women of reproductive age (15- 49 years) and men (15- 60 years) who were selected with the support of community leaders and service providers in both refugee and host communities. This study was conducted in the refugee-affected district of Adjumani, West Nile region of Uganda in the three settlements of Pagirinya, Nyumanzi, and Mirieyi and surrounding host communities. The three settlements were purposively selected. Two of the settlements (Pagirinya and Nyumanzi) have the highest number of refugees and have existed in the district for over 23 years. Whereas Mirieyi settlement has fewer number of refugees, and it is the newest and is located near Adjumani town. The settlements are far apart and are spread throughout the district. Mirieyi settlement is 7 km away from town, Nyumanzi and Pagirinya are each 27 kms away from Adjumani town. The rest of the settlements are located 15 to 42 km from the town center with very poor road network, a lack of public transport except for the very expensive private boda-bodas (motorcycles). Adjumani district was purposefully selected because it hosts mainly the refugees from South Sudan whose number (214,453) are almost the same with the host population (214,377). The refugees have diverse ethnic backgrounds including the Dinkas, Kuku, Nuer, Kakwa, Madi, Siluk, Acholi among others. They speak the central Sudanic language of the Nilo Saharan language family. The indigenous people speak the Madi, Kuku, Acholi and Lugbara languages that are almost similar with that of the refugees.

### Sampling and recruitment

Purposive sampling approach [30] was employed to select participants for all the qualitative data collection methods (KIIs, IDIs and FGDs) to ensure inclusion from both gender, different responsibilities in the district/community, religion, educational level, duration of stay in the area and refugee/host status for atleast one year and above. Participants excluded in this study were those not registered as refugees, arrivals of less than one year in the settlement, critically ill (not able to do basic life activities without support of a carer). From the host community, all those who have not lived in the community of study for atleast one year and may be critically ill. Overall, 220 participants were recruited, 112 (51%) were females and 108 (49%) were males. All participants were aged between 15–60 years. Eleven key informants (8 males and 3 Females) were selected and interviewed. These included OPM representative (1), health workers (2), religious leaders (2), cultural (1), community (1), district leaders (3) and refugee leader (1).

#### Data collection

A total of 6 Research Assistants (RAs) selected from a pool of experienced persons from the district and Makerere University School of Public Health participated in data collection. Some of the RAs were social scientists with master’s degrees and were able to speak the languages best understood by refugees in the settlements and the host communities owing to the ethnic diversity. This helped to reduce on the need for interpretation that would have breached confidentiality. The commonly spoken languages included English, Dinka, Madi, and Arabic. The RAs were trained on qualitative research methods and how to use the tools. Data were collected using translated and pre-tested interview guides that were developed based on previous studies and adapted in relation to decision-making [31].

Pre-testing of the tool was carried out in Agojo settlement and its host community in the same district. Interviews were conducted in secure and private places that were convenient to participants to ensure privacy. Open-ended questions were used to enable the respondents tell their stories on the decision-making processes.

Audio-taped interviews were transcribed verbatim and translated into English. The Principal Investigator checked all the transcripts to ensure accuracy, completeness, and consistency. Data were saved as Word documents under password-protected files with names showing the type of data collection method, location, date of data collection and data collector numbers. We generated codes using interactive and repeated process until when no more codes and categories was emerging. The codes were then organized into sub-categories, categories, and broad themes. Sub-categories developed guided the development of themes and descriptive quotes identified [32]. Thematic analysis was conducted following procedures described by MacQueen [33].

Overall, forty-nine (49) IDIs were conducted with females and males of 15–19 years, females, and males of 20 years and above (see Table 1). Twenty-seven were refugees (13 males and 14 females) and twenty-two were from host communities (7 males and 15 females). We conducted 20 FGDs consisting of 8 participants each (160 participants). Since this study involved different age groups, we stratified them into four different categories to enable homogeneity and free expression of ideas under discussion. Females of 15–19 years and 20 years and above, males of 15–19 years and 20 years and above.

**Table 1:** Socio—demographic characteristics of all participants

Variables	Refugees population		Host population		Total population
	**108** (49%)		**112** (59%)		**220**
	**Number**	**%**	**Number**	**%**	**Total, [%]**
**Gender**					
Males	55	50.9	54	46.4	**109 (49.5)**
Females	46	49.1	65	53.6	**111 (50.5**
**Tribe**					
Madi	8	28.1	22	97.3	141 (62.4)
Dinka	14	43.0	0	0.0	49 (21.7)
Nuer	4	12.3	0	0.0	14 (6.2)
Ding Dinka	2	7.9	0	0.0	9 (4.0)
**Marital status**					
Married	21	8.8	16	2.7	13 (5.8)
Single	4	80.6	7	81.3	178 (80.9)
Separated	2	8.3	1	12.5	23 (10.5)
Widowed	0	6.5	0	5.4	13 (5.9)
**Education**					
No education	5	4.6	0	0.9	6 (2.7)
Primary	10	12.0	5	1.8	15 (6.8)
Secondary	10	51.9	15	55.4	118 (53.6)
Tertiary	2	27.8	2	28.6	62 (28.2)
**Occupation**					
Housewife	17	8.3	13	14.3	25 (11.4)
Formal worker	0	72.2	6	52.7	137 (62.3)
Business	6	0.0	0	12.5	14 (6.4)
Student	4	24.1	3	31.3	61 (27.7)
**Religion**					
Catholic	14	3.7	19	3.6	8 (3.6)
Anglican	10	80.6	1	54.5	148 (67.3)
Muslim	0	9.3	1	16.1	28 (12.7)
Others (Born again, SDA)	3	7.4	1	21.4	32 (14.5)
**Age range per method**		2.8		8.0	
**FGDs**	**72**	**45**	**88**	**55**	**160**
**Age**					
15–19 years	40	55.6	24	27.2	64 (40.0)
20–30 years	16	22.2	32	36.4	48 (30.0)
31–40 years	16	22.2	32	36.4	48 (30.0)
41–50 years	0	0	0	0	0
51–60 years	0	0	0`	0	0
**IDI**	**27**	**55.1**	**22**	**44.9**	**49**
**Age**					
15–19 years	7	26	2	9.1	9 (18.4)
20–30 years	6	22.2	8	36.4	14 (28.6)
31–40 years	10	37.0	11	50.0	21 (42.8)
41–50 years	4	14.8	1	4.5	5 (10.2)
51–60 years	0	0	0	0	0
**KII**	**2**	**18**	**9**	**82**	**11**
**Age**					
15–19 years	0	0	0	0.0	0 (0.0)
20–30 years	0	0	1	11.1	1 (9.1%)
31–40 years	0	0	1	11.1	1 (9.1)
41–50 years	1	50	4	44.5	5 (45.4)
51–60 years	1	50	3	33.3	4 (36.4)

Nine FGDs were conducted with refugee groups (5 males only and 4 females only), of these, five groups were with young people aged 15–19 years (3 males and 2 females). Eleven FGDs were conducted with the host population (5 males only and 6 females only), of these, three groups were with young people aged 15–19 years (1 male and 2 females). In every FGD, there was a facilitator, a note taker, and one who recorded and assisted to moderate. The FGDs lasted between 45 and 60 min, while the KIIs and IDIs lasted between 30 and 40 min. The interviewers started by explaining what it means to decide a way of motivating participants to speak more freely [34]. Interviews were conducted until saturation was reached. Following the procedures recommended by [35], the facilitator and the Research Assistant met immediately after the focus group discussions to debrief on the session and compared notes. They discussed additional information needed based on the emerging themes, any salient issues, and the general impressions of the discussions.

### Data management and analysis

**Fig. 1:**
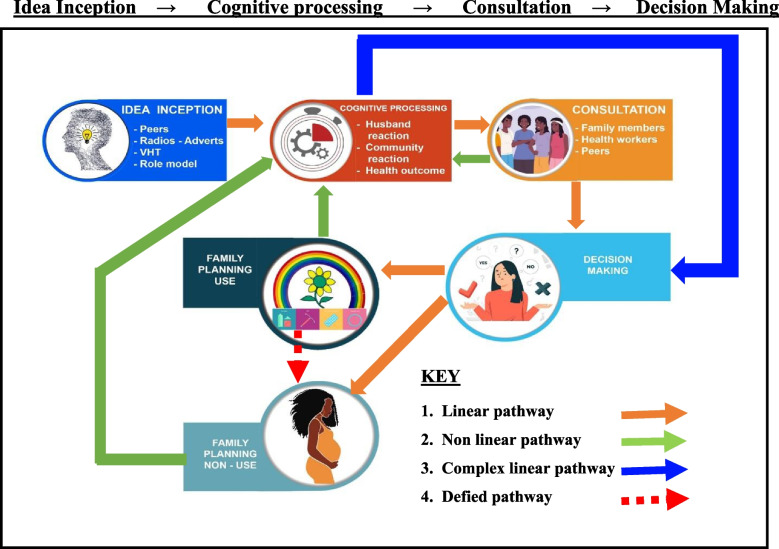
Interlinkages of the decision-making process for contraceptive use; pathways for decision making

All audio recordings from the FGDs, KIIs and IDIs were labeled, transcribed verbatim, and translated into English by an experienced research team who were fluent in Madi and Arabic as the main languages and the recordings stored. Data were analyzed over 3 a month’s period to generate codes, sub-categories, categories, and Themes. To ensure reliability and validity, inductive, team-based coding was done by three research experts led by the Principal Investigator (PI) [36]. They read the transcripts, discussed emerging issues, and agreed on common themes. A codebook was developed following Braun and Clarke’s procedure [37]. This process of coding was repeated interactively until no more codes and categories could be generated due to data saturation. While coding, there were interactive discussions arising from the discrepancies and disagreements that were resolved by having same meaning of the codes before moving to next the step. Data were coded using Atlas ti. version 14 and analyzed using content analysis because of its credibility, transferability, and dependability. A constructivism approach of constructivist ontology and epistemology approach was employed [38]. This dynamic and complex four-level model was developed, demonstrating the pathways followed for decision-making. It has enabled us to develop a theory with interlinkages to the decision-making processes for contraceptive use (Fig. 1). The pathways included the inception of the idea, the cognitive processing of the idea, consultation of significant others, and making decisions about the outcome of using contraceptives or not.

### Ethics statement

Ethical clearance was obtained from the Makerere University School of Public Health Higher Degrees Research and Ethic Committee (HDREC) registration #188 and Uganda National Council of Science and Technology (UNSCT) Registration #SS809ES. Written permission to access the refugee community was obtained from the Office of the Prime Minister (OPM). Participants were informed about the purpose of the study. The participant’s rights and confidentiality were guaranteed [39–41]. Participants were informed of their voluntary participation in the study and were free to answer all questions or not. They were also free to withdraw at any time without any consequences. Their written consent was obtained before the commencement of the interviews. Written consent was obtained from parents/guardians on behalf of the adolescent participants who were in the age category of 15–18 years [42].

## Results

The presentation of the result of this study has been done using the consolidated criteria for reporting qualitative research (COREQ) [43]. The construction of the final decision-making theory involved an interactive process. Codes were generated from raw data and synthesized. The study found that decision-making processes entailed linear and nonlinear internalized cognitive and contextual processes with four dynamic pathways. We explored decision-making processes for contraceptive use or non-use by refugee and host communities involving various factors that were coded to have influenced the final decision. These also informed the sub-categories upon which the participants based their judgement in relation to personal fears, social influence, health concerns, economic difficulties, and the health benefits of contraceptive use. These formed the categories, leading to the themes that included four dynamic pathways (see Fig. 1).

It was noted that all participants expressed conceiving ideas about birth control from various sources. They went through the phase of internalized cognitive thinking and made several considerations on how to proceed. For the women who followed the linear pathway, they started with 1) idea inception, then followed through to 2) cognitive processing, 3) consultation, and 4) decision-making to either use or not use a contraceptive. The complex linear pathway was followed when participants did not go through consultation but straight to decision-making after internalized cognitive and contextual considerations. However, participants who followed the non-linear pathway continuously went back to cognitive processing. Some women after consultation, or those already using and those not using contraceptives, decided to go back to cognitive processing to reconsider their earlier positions. This study found that some women who started off not using contraceptives ended up using, while some who started off using contraception ended up dropping out due to the dynamics involved in the various stages. This was either due to personal experiences of using or not using contraception. All these stages are pertinent to individuals that required nurturing by giving accurate information for women and men to make informed decision.

The common finding was that after a thought process, women from both communities would go ahead to consult their significant others. Based on the outcome of the individual consultations, the women decided whether to use contraception or not. Others would not consult but would make decisions after thinking about the idea. It is worth noting that all stages were very important and built on each other for the complete process of decision-making as seen in Fig. 1 of the constructed model for decision making processes. However, there were some categories of women whose contraception status was changed through violence. Thus, their partners forcefully recommended removal of the contraception where a Long Acting and Reversible methods like implants or IUDs was being used. Participants reported that some women experienced extreme violence leading to their death because of using a contraception.

### Comparison with the ethical decision-making model

When this study was compared with the ethical decision-making model, the difference was that the decision-makers considered a fixed model ascribed to them to assess the impact of their decision as opposed to our study findings where the participants made decisions based on the complex processes which may be a mixture of irrational and rational decisions. This approach involving complex processes helped to appreciate that decision-making gives the opportunity for one to go back to any stage and rethink the new direction as opposed to the integrated ethical decision-making model that focused on the “person-situation interactionist approach” [44]. This study therefore applied the inductive approach of using data collected from participants to construct a model for decision-making (Fig. 1). These processes are further elaborated in detail below.

### Idea inception

Twenty-nine (29) participants from refugee populations (KII = 2 and IDI = 27) and 119 participants from the host communities (KII = 9 and IDI = 22) reported that the process of deciding to use contraception started with the inception of the idea. This was further supported by participants from both populations during the 20 focus group discussions (FGD = 9- refugees) and FGD = 11-host) that idea conception is the first step they went through when deciding to use a contraceptive. They mentioned that it was instigated by the felt burden related to family size, individual health concerns, and external influences from peers, adverts from radios, Village Health Teams (VHTs,) and role model families.

Majority of the participants mentioned that the family size exerted pressure on the family welfare leading to poor living conditions and hardships that triggered the idea to think about considering contraceptive use. Most of the women reported that meeting the basic needs of a large family like education, health care, and nutrition was expensive and burdensome. They added that this burden was mainly relegated to them as the men focus on their social life including alcoholism. Young people also mentioned economic hardship, concurring with the idea of decision to use contraception for child spacing.

*“…when you ask them, they say it’s problems that make them come for contraceptive services, so, that they at least want to space, such that they overcome those problems like being unable to afford better education, shelter for the children, good clothing yeah all those…”* **(**KII – service provider 20+_Health Centre -Adjumani).

And,

*“…me right now if I produce many children ehhh and am still young with nothing almost and my family is also very poor, I cannot produce more children and that is why I have stopped with two children, and I am encouraging my peers to use contraception...”* (FGD - male 15-19 years_ Settlement)

Additionally, the participants reported personal health concerns such as the risk of frequent childbirth, advanced age and previous complications as reasons for conceiving the idea of contraceptive use. Exposure to information from radios, peers, role model families, and the outreaches by implementing partners triggered the idea of contraception.

*“…yes, I got contraceptive information from partners but the fact that I got pregnant within six months of my previous childbirth, I considered the idea to use contraception…”* **(**IDI- Female 20+_Settlement).

And,

*“…but when you go into our houses who starts to think about contraception? It’s the wife who comes up with the idea because she is the one who gets pregnant and experiences the challenges related to pregnancy. So, when they give birth, they need to space...”* (KII male 20+_Host community).

### Cognitive processing

Following conceiving the idea about contraceptive use, participants went into cognitive processing which was informed by internalized thought process considering personal perceptions, fears and individual circumstances. Cognitive processing also entailed consideration of external contextual factors. According to most participants, several internal factors were considered including myths and misconceptions around contraception such as infertility, low libido, abdominal pain, cancer, and giving birth to a deformed baby. In some instances, these generated unanswered questions on whether to go for contraception or not. Some participants reported having to consider the potential reaction of their husbands, parents-in-law, peers, cultural and religious leaders who were against contraception and the likelihood of the husband marrying another wife.

“…*if you are very weak, your husband will chase you away from that home when found using contraceptives…”* (IDI female 20 + _Settlement).

Although most of the male participants were against use of contraception, some young male participants particularly from the settlements reported that women need to plan for the next baby for their future benefit.

*“…educated and working women in office who have at least one child, don’t want to get pregnant for fear of interrupting their work. So, to me, for a woman to succeed in life, she needs contraceptives to space and only get the next child after planning. This is beneficial to the women of this generation …” (*FGD male 15-19_Settlement).

Most of the participants from the refugee population were more concerned about where to start in case they made the decision and how their lives would be while on contraception. Depending on individual considerations, and the experiences from what they saw with model families, some participants reported going straight to decision making stage while others went through the consultation stage with their significant others.

*“…what encouraged me to make decision to use contraception was due to the practical experience I saw from the model families who use contraceptive methods and how they always feel better. There are no quarrels amongst the couples, their children are few and manageable and they love each other…”* (FGD_ female 20+ Host community).

### Consultation

Participants who had unanswered questions from the cognitive processing stage reported going on to consult their significant others. These included immediate family relatives, friends, health workers, parents, and peers who were either using or not using modern contraceptives. Some participants reported that the main issues of interest were consent and information. Some women followed the linear pathway and others followed the nonlinear pathway. Those who followed the linear pathway went through consultation with their significant others and eventually to decision-making. Whereas some women reported having to consult their husbands and mothers-in-law to secure their approval for contraceptive use. This was also done to avoid the likely occurrence of violence if they get to know. Some women consulted and were reluctant to take up contraception but were forced by their significant others to start on a method given their situations of frequent childbirth, thus were taken to decision-making stage by force.

*“…when I consulted my mother, she encouraged me to start using contraceptives because, at 18 years, I gave birth to my first born. In that same year, I gave birth to a second baby. I again conceived a third child when the second one was 9 months. She then encouraged me even when my husband had refused…*” (IDI _Female 20+_ Settlement).

Others reported that after consulting, they would go back to the cognitive processing stage to think about the information, the advice, and the fears including threats from their partners before they could decide. However, overall, participants reported the usefulness of consulting significant others, and the availability of services that played a big role in giving them information to move to the stage of decision-making. Some women also had several unanswered questions about the choice of methods, service delivery points and side effects. They reported seeking information from peers, health workers, Village Health Teams (VHTs), and role model families to get clarity on their unanswered questions. While the VHTs are key players in promoting contraceptive use because they advertise it amongst the community members and physically escort the women to the hospitals for services, the male role models also inspire fellow men to use contraception for improved livelihood.

*“…during our intervention here in the settlement, we created what we call, the male role models. These are men who are supposed to go and preach about the use of contraceptives to fellow men to relieve the women from the risk of frequent childbirths…” (*KII_Male 20+_Settlement).

Also, some women went back to the cognitive processing stage after consultation given the type of information they received from their significant others. Where the information was not sufficient to enable decision-making, there was a relapse back to think through the idea once more and followed the linear or nonlinear pathway to decide to either use or not use contraception.

*“…I also have a bad view about contraception because what happened to my brother’s wife was bad. It compelled me to use natural method but if I get more information, I will take it up in the future*…” (IDI_Female 20+_Settlement).

### Decision making

At the stage of decision-making, it was clear that no woman remained permanently at the previous stages without deciding. While they went back and forth several times to think through and consult repeatedly, they still landed at the stage of decision-making. This is because of the dynamics related to the interplay between the internal and external environment. Three different levels of decision-making were considered for contraceptive use. Some got permission officially to use contraception. Others who made a personal decision without seeking permission and some were denied permission but went ahead to use contraception regardless of what their significant others advised.

*“… in our culture as Dinka, the use of contraception is an abomination. However, if I want to use it, I don’t need to tell anyone. I simply do it with my service provider and avoid any discussion even with my close friend or fellow woman. If the information leaks to them, they will tell your husband or in-laws and problems will come up, so just do it secretly and alone…”* **(**IDI_Female 20+_ Settlement).

But also, there were women who upon conceiving the idea, went through cognitive processing and straight to make the decision to either use or not use contraception without involving any external parties. For the women who made the decision to use, it could have been due to accurate information received at inception and/or the ability to make personal decisions. They reported using contraceptives stealthily to avoid quarrels that would arise should the husbands get to know. There were also categories of women who had made the decision to use but experienced some challenges and went back to cognitive processing. The research participants reported facing several challenges related to side effects, lack of a method of choice, harassment by their husbands, and negative attitudes towards contraception by men and some community members.

*“… some women are neglected by their husbands without any social support. This will also cause hatred between wife and husband in the homes causing unnecessary tension and worse of all, separation where the husband may chase away the wife from his home due to anger...”* (IDI_Female 20+_Settlement).

And,

*“...I did not discuss it with my partner because he was always negative about contraception and because of fear of quarrels and family disintegration, I just started on a method quietly and he is not yet aware…”* (IDI_Female 20+_Settlement).

While the study participants acknowledged that contraceptives have health benefits that come with it, nearly all participants from IDIs (48/49) confirmed that the benefits are mainly for both mother and baby. Therefore, based on this information, they made their own decisions. However, they asserted that personal decision is only possible for women who are economically empowered and can sustain themselves and provide basic needs should the husband discover them with contraceptives.

*“… I decided to use implant because I have two children now in a period of less than 2 years. After giving birth to the first child, six months later I got pregnant with the second baby which made me decide to go for a long-term method of contraceptive to get time to do some business to support them…” (*IDI female 20+_Settlement).

Some women took up contraception with consent. Research participants especially from the host community reported getting consent and support from their husbands to start contraception. They added that due to the joint decision, they have enjoyed the support and do not have any challenges and no side effects. The male participants also re-affirmed the need to support the women’s decision to use contraceptives.

*“… because of joint decision, I used pills for spacing, and when we were in Antenatal with the second pregnancy, we were doing very well, having good nutrition, and doing a lot of good things together. There were no challenges and no side effects that I ever experienced with pills as I was taking it daily without fear of anything…”* (IDI- Female 20+_ Host community).

Furthermore, some women had fears related to being chased away from the family through separation, physical beating, being called a prostitute and adding a co-wife. As such, they said the best option is to use contraceptives quietly and avoid sharing even with the closest friend for safety. This was re-affirmed by male participants who alluded to this saying that the power for decision-making lies with them and not the women.

*“… if my wife decides to use a contraceptive without my decision, this will lead to an automatic fight and even separation. I will call my brother to come and get a solution to this problem and chase her away...”* (FGD_Male 20+_Settlement).

Although some women wanted to use contraceptives, they had fears due to reprisal and community judgment. The community looks at women who use contraceptives as prostitutes. Men who learned that their wives were on contraception, retaliated in various ways which scared some women from making the decision to use contraception.

*“… many women are aware of contraceptives but are afraid because men are against it. The consequences are undesirable and fatal in some cases. Last year, 2 women were hacked to death in one Sub County because they were found using contraceptives without their husband’s consent. This has created a lot of fear among Madi women in Adjumani. And those women who use contraceptives, do it at their risk...”* (IDI, Female- 20+ _Host community).

Also,

*“…one of the factors is cultural and environmental as a hindrance in the society. If the community gets to know that you and your partner have gone to Nyumanzi clinic and got contraception services, community members will start gossiping around saying your partner is a prostitute. That is why some families have decided to continue producing children…”* **(**FGD_ Male 20+ _Settlement).

There was also a category of women who after making their own decision to use contraception, the partners forced them to discontinue. Some participants particularly from the refugee community reported that upon discovering that they had any method, both the woman and the health workers face the wrath of the husband. Many of the male participants confirmed that they have always fought with their wives for deciding to use contraception without their consent.

*“… women make decisions for contraception because men have failed to play their roles. If I failed to make decisions and my wife goes ahead to use contraception, fights will ensue in the form of showing my authority until its removed. I will call my brothers to come, report to them the issue, emphasizing that it was done without my consent and their advice thereof shall be followed immediately...”* (FGD_Male 15-19_Settlement).

## Discussion

This study brings new insights and a deeper understanding of the cognitive processes involved with the external environment for contraceptive decision-making. The study found that participants from both populations, refugee, and host and across all the different methods of data collection (IDIs, KIIs, and FGDs), followed dynamic stages with linear and nonlinear pathways to decision-making for contraceptive use. Many of the women largely followed the linear pathway from conception, cognitive, consultation and decision-making for contraceptive use.

### Idea conception

This study revealed that during the inception stage, the ideas were conceived because of information acquired from various sources that included the Village Health Teams (VHTs), role model families, peers, adverts on the radios and community outreaches. The participants reported that they considered the experiences of their peers to determine their next action. This means that the traditional health care of waiting for clients to go to health facilities to seek information can no longer suffice because they exchange information amongst themselves. However, given that some peers did not have accurate information, it did not appropriately facilitate the thinking process but continued to the next stage of the cognitive process. A similar finding in Kenya revealed that limited information is one of the barriers to contraceptive use that requires caution in order to influence decision to use [45]. This has implications for public health in that any wrong information received by potential users may take a lot of effort to undo and may deter them from considering contraceptive use. The study revealed that, VHTs did not only conduct door-to-door visits to give information on contraception but also organized community sensitization and linked clients to health facilities which has enhanced referrals because of trust by the community. This finding is similar to a study done in Nigeria that confirmed that community health workers have a big stake in increasing contraceptive information and use [42]. Based on the above, we recommend that the government and partners intensify health promotion campaigns based on credible sources of information at community level.

### Cognitive processing

This study introduces the internalized invisible cognitive processes involved in decision-making for contraception. The research participants reported that before considering any decision, they had several factors that influenced their action. Thus, the pathway followed was determined by either positive factors such as health benefits, social support, women empowerment, gender roles, and economic situation or negative factors which included men’s negative attitudes, Social cultural norms and beliefs, myths and misconceptions, side effects, limited information, gender disparities and prices paid for using contraception. Furthermore, this paper argues that decision to use a contraceptive is not spontaneous except in circumstances of duress. We observed complex and iterative processes involving internalized cognitive processes and factors within the immediate personal and distal environment. This study concurs with a study findings in Malawi and Afghanistan which revealed that the immediate personal and distal factors such as cultural and religious norms, myths and misconceptions, disapproval by partners, limited women empowerment and side effects affected contraceptive use [46, 47].

Some participants reported being afraid of getting cancer, fibroids, infertility, and abnormal children borne from contraceptive use. These myths and misconceptions were established from both populations who confirmed that it is a big hindrance to decision-making for contraceptive use. This is likely because of limited information by some significant others consulted and as such, do talk on hearsay without facts. This finding concurs with a study done in South Africa that also found that rumors impede access and utilization of contraceptives [48]. This is probably due to the societal setup where women discuss issues about their reproduction no matter the information shared. This has implications on public health in that the more propaganda on contraceptives that is spread around the community, the more women and men may continue rejecting the services. Community dialogues should be organized to address those myths and misconceptions and give accurate information about contraception.

### Consultations

Contraception is beneficial for both health and socio-economic reasons which include birth spacing, reduced unintended pregnancies, prevention of unsafe abortions, improved maternal and child health, prevention of sexually transmitted diseases, and engagement in educational opportunities and economic activities to reduce poverty [49, 50]. This study revealed the widely held view that there are good things that come with contraceptive use. Many participants reported admiring the way the role model families lived a happy life. This study revealed that the host community was more aware of the health benefits of contraception compared to refugees. Whereas among the refugees, the study established the limited promotional materials with unclear sources of information leading to receiving inappropriate and distorted contraceptive messages from elders that started back home in South Sudan. A similar study conducted in South Sudan revealed that there were limited contraceptive promotional efforts by the South Sudan authority [51]. This was backed by another study conducted in Ogun state in Nigeria which established that source of information is key and when that source is not well trained, may distort the very good intention leading to low uptake [52]. In South Sudan, this could probably be due to conflict that has destroyed the infrastructure leading to fragmented communication channels in the community and limited access to contraceptive information and services. Whereas, a study done in Malawi revealed that social support increased the chances of decision making for contraceptive use [46]. Considering that inter personal communication helps women to discuss their reproductive intentions, we recommend that more role model families be trained and significant others sensitized to respond accurately with contraceptive information when consulted by potential users [45].

### Decision-making

Although some research participants reported that an economically empowered woman can make personal decisions if she can sustain herself with basic needs, it was not obvious to the majority because they still had fears of the implications if their husband found them using contraception. This study established that some women from the refugee population were more confused at the decision-making stage due to fear of possible reaction from the external environment. However, we found that women from both communities who engaged in small income generating activities or had tertiary level of education considered themselves empowered and were able to make their own decision to use contraception without consent from significant others [53]. On the other hand, even with women’s empowerment, this study still found that some participants did not have decision-making power and as such were not able to make a personal or joint decision with a partner. This finding concurs with studies done in Afghanistan and Nigeria which revealed that women were empowered due to the available opportunities to discuss their health issues [54, 55]. Given the above findings, there is a need for the government and partners to understand this complexity and design a comprehensive community intervention involving men to promote equitable access to contraceptive services.

### Dynamics of contraceptive use

Although some participants reported that contraception is good for the mother, baby and the general well-being of the family, many respondents from the refugee community are still challenged with major barriers of socio-cultural norms and beliefs such as the need for large families, girls considered for bride price, replacement of lost family members, promiscuity and negative attitudes of men and gender disparities. Therefore, there were deliberate efforts by significant others (elders) who were antagonizing contraception programs, saying it would kill their families and as such, encouraged their people to produce many children who will replace the lost family members and be a source of security, free labor, and wealth. This finding concurs with several studies which revealed that cultural and religious norms including social stigma were significant factors affecting contraceptive use [54, 56–59]. This has implications for public health as culture take precedence to the health of women and girls. Considering the above findings, we recommend that contraceptive programming should involve everyone in the community for equitable access to information including cultural/religious leaders and other ways of making money as opposed to focusing on girls who after all, will take some time to grow before marriage.

This study revealed that the phenomenon of men being against contraceptive use was more common among the refugees compared to the host. This study revealed that men think that their wives go for contraception because they don’t get sexual satisfaction. They alleged that women go to other men for satisfaction and use contraceptives as a cover-up. On the other hand, the study also revealed that many women say that what made them use contraceptives against their husband’s will is due to their negligence. Some women reported that most men engage in drinking alcohol, leaving the entire responsibility of family welfare to them. This could be due to limited exposure by men to contraceptive information and as such, do not agree with the practice. This finding concurs with a study done in India which revealed that men’s negative attitudes have affected women’s access to contraception [60]. This is probably due to the male dominance that women must consult on all matters of the family. This has public health implications on women who may remain in a state of ill health as they wait for authorization by the partners and yet contraceptive services would help to avert unsafe abortion that comes due to unintended pregnancies [61, 62]. Designing behaviour change communication targeting men and women could help to change their attitudes towards contraception.

This study also revealed numerous health concerns associated with contraceptive use. The research participants from both populations were concerned about the side effects which often resulted in either discontinuation of use or switching a method. The research participants cited low libido as a common occurrence among the host population as opposed to refugee population. This finding concurs with studies done in Kogi state in Nigeria and New Zealand, which revealed that side effects affected the decision for contraceptive access and utilization [63, 64]. This is due to the misleading information on contraception programs that are regarded as a threat to a reduced population, leading to rising deterrence. This has implications for public health in that many families may listen to the propaganda at the expense of their lives and the health outcome of their children. We, therefore, recommend that while health education is carried out for the community to clarify the health concerns, there should be a deliberate effort by the government to build the capacity of service providers to appropriately manage side effects.

In this study, gender disparities were common and affected women’s decision-making ability to use contraceptives [65, 66]. In most African societies, men are the only decision-makers whose powers are not questionable no matter the circumstance. This has been in existence for a long time and indeed no one can dare question this unprescribed authority. We found that some male participants expressed concerns that their wives never consulted them before deciding to use contraceptives which they said, undermined their masculinity and authority of being the head of the family. Therefore, men will always exercise their powers over their wives in decision-making for contraceptive use. Based on the notion that men want to control the decision-making powers of women for contraceptive use, there is a need for government and key stakeholders to scale up the male involvement strategy in health programs with a focus on contraception.

Although women were aware of the benefits of contraception, they were overtaken by the fear of implications. The women were in constant fear of what their husbands would do when discovered to be using contraceptives. As discussed earlier, the fear of perceived violence against actual violence is a price that women pay and as such influences the type of decision made for contraception. We further established that many women who made a personal decision to use contraception and were discovered, paid a price that ranged from neglect, beating, rejection, and divorce/separation to the husband marrying another wife. Paying bride prices was regarded as normal among the refugees compared to the host and as such, the men could marry more wives to discipline the current wife. This is likely because the refugee community has a quest for large families. This finding concurs with studies done in Afghanistan and Kenya [47, 67] which revealed that domestic violence manifested in different forms in women who used contraception. It is probably due to the community’s perception of contraception programs that are regarded as a threat to the reduced population and limited knowledge about women’s right to health. This has implications for public health in that many women may continue to pay the price for using contraception while putting their life in jeopardy. We recommend that government may embark on rigorous health education drives that will address intimate partner violence, gender disparities, and social norms related to contraception. We also recommend strengthening male involvement in contraception programs as champions and promoting positive masculinity including discussing issues of fundamental human rights to health by all as enshrined in the Uganda constitution of 1995.

This study also established enablers to decision-making for contraceptive use. Research participants confirmed that the pinch of no resources was more compelling among both populations and as such have reconsidered their fertility intentions. They attested to the fact that the role model families have demonstrated a good life for a manageable number of children. The participants reported poor quality of life and lack of funds to pay for the tuition of their children in good schools, remaining with poor quality education and poor quality of life. This finding concurs with a study done in Tanzania [68] which revealed that household responsibilities influenced the use of contraception. This is due to the pain the women go through in caring for children’s welfare and supporting a family amidst economic hardships. This has implications for public health related to poor health outcomes. We therefore, recommend that both men and women be targeted with a comprehensive contraception program that will create more awareness in the community to harness joint decision-making.

Women empowerment was cited among both populations who confirmed that educated and working or businesswomen embrace child spacing because they wanted to concentrate on their jobs/business. In this study, we also found that income-generating activities have empowered some women and as such have decided to use long-term methods that provided them with longer protection. This category of women made the decision regardless of the external threats. This is likely because of the ability to provide for their social needs. Secondly, due to small grants given to female refugees as a start-up kit for financial support and as such, able to support themselves and confidently go for contraceptive services without waiting for spousal consent. This study concurs with finding by [69] which revealed that women who are empowered do not fear to make decision to use contraceptives.

### Perceived unsustainable cultures

Given the uniqueness and fragility of the humanitarian context, refugees are faced with multitudes of challenges that need to be considered before planning contraceptive programme. The context of refugees back home in South Sudan was so conducive to enforcing the cultural norm of a man going away with cattle to graze in another community for 2 years before returning home. When a woman gives birth, the husband will not have sexual intercourse with her until the baby is 2 years. In some instances where the husband cannot go away, she sleeps in the house of her mother-in-law to avoid any possible contact with her husband. But because of the challenges that came with conflict, the refugees in Uganda are confined in one place with limited movement and therefore, have no options of going far away as the wife recovers and breastfeeds for 2 years. There is a break in this tradition because the women get pregnant before the ascribed 2 years as they continue to share a room with their husbands.

Despite the numerous efforts to increase contraceptive use this study still revealed that refugees don’t want to use contraceptives because they have their own natural family planning methods which unfortunately is challenging to practice when in the settlement. This concurs with various studies by [47, 59, 60, 70] who revealed that natural family planning is preferred by many tribes from South Sudan.

### Reflexivity

Rapport was built with participants before any questions were asked to ensure that trust is obtained from the researchers since contraceptive use is a sensitive issue among refugees. The researcher though knowledgeable about the subject matter, assured participants that whatever discussions were held with them were for the study and nothing else and that the findings were intended for the improvement of contraceptive use among refugees and host populations in Adjumani [71, 72].

### Human rights issues

Human rights-based issues came out strongly and evident that the community could not differentiate between human rights and the possession of wives. Research participants from both communities asserted that upon paying the bride price to the girl’s family, she automatically becomes the property of the man. Although, the United Nations declared that contraception is a human right that is upheld by many other rights as an intrinsic right, the male participants in this study seem ignorant about it. They asserted that contraception is not a human right but it's one’s desire and that is the reason why they do anything to women who are found using contraception without their consent including killing them [73].

Women are viewed as property among both refugees and host population because of the dowry. This concurs with a study that revealed violent death due to jealousy stemming from contraceptive use without consent from a partner [74]. This has public health implications in that, innocent women due to fear, cannot access health and as such end up with poor health outcomes or death. Therefore, it is imperative to sensitize men about the human rights-based approaches to health to appreciate that every human being has a fundamental human right to access health without coercion or denial.

## Conclusions

Decision-making is considered as an important phenomenon that every individual undertakes in everyday life. Contraceptive use is understood as a cognitive and behavioral outcome of a complex decision-making process. This study showed dynamic decision-making processes involving complex linear and nonlinear internalized cognitive and contextual processes with four pathways (inception, cognitive processing, consultation and decision-making). Based on the above findings, government and partners need to understand the complexity involved in decision-making for contraceptive use and ably address it if the contraceptive prevalence rate is to be improved in the humanitarian setting and the host communities in Uganda. Furthermore, interventions to increase contraceptive use should target both users and significant others who influence the decision for contraception particularly among refugees.

### Study limitation

This model was developed based on the perspective of participants living in the humanitarian context in Uganda and using a qualitative research approach. This may not apply to other humanitarian settings in low- and middle-income countries. Future studies using mixed research methods may be needed to explore the research issues from different epistemological and ontological positions.

## Data Availability

The datasets used were analyzed during the current study and are available from the corresponding author on reasonable request.
